# Asiatic acid ameliorates tubulointerstitial fibrosis in mice with ureteral obstruction

**DOI:** 10.3892/etm.2013.1197

**Published:** 2013-07-02

**Authors:** CHANGGENG XU, WEI WANG, MINGWEI XU, JIE ZHANG

**Affiliations:** 1 Department of Urology, Renmin Hospital of Wuhan University, Wuhan, Hubei 430060, P.R. China; 2 Huangshi Central Hospital, Hubei Polytechnic University, Huangshi, Hubei 435000, P.R. China

**Keywords:** asiatic acid, renal fibrosis, TGF-β1

## Abstract

Asiatic acid (AA) is one of the triterpenoid compounds present in *Centella asiatica* and it has been shown to be capable of attenuating liver fibrosis. In the present study, we investigated the effects of AA on renal tubulointerstitial fibrosis in mice with unilateral ureteral obstruction (UUO). Mice were divided randomly into five groups (n=5 per group): the sham-surgery (Sh), UUO plus vehicle treatment (UUO+V), UUO plus 1 mg/kg body weight AA treatment (UUO+A1), UUO plus 4 mg/kg body weight AA treatment (UUO+A2) and UUO plus 16 mg/kg body weight AA treatment (UUO+A3) groups. The mice were treated with AA daily by oral gavage from the day subsequent to surgery for six days. On the seventh day, the mice were sacrificed for examination. Tubular injury was observed in the renal cortex of the mice administered the vehicle, while high doses of AA were observed to exert a significant suppressive effect on tubular injury. Interstitial fibrosis, increased expression of α-smooth muscle actin (SMA) and transforming growth factor (TGF)-β1 and phosphorylation of Smad2/3 were induced by ureteral ligation; however these effects were abrogated by intermediate and high doses of AA. These results suggest that AA may ameliorate tubulointerstitial fibrosis by reducing tubular injury, fibroblast activation and extracellular matrix (ECM) accumulation mediated by Smad-dependent TGF-β1 signaling.

## Introduction

It has been estimated that up to 15.2% of the adult population exhibits a certain degree of chronic kidney disease (CKD) (1), while the prevalence of patients with end-stage renal disease (ESRD) has shown a global increase. CKD leading to ESRD is associated with tubulointerstitial fibrosis, regardless of the underlying causes (2). Interstitial renal fibrosis is characterized by tubular atrophy, lumen dilation, accumulation of fibroblasts and increased interstitial matrix deposition (3). While the understanding of the molecular mechanisms of fibrogenesis has improved, only a limited number of antifibrotic therapies are currently used in the clinic (4). Therefore, further investigations into specific treatments to target fibrosis are required.

Although most types of resident and bone marrow-derived cells are involved in fibrogenesis, fibroblasts are considered to be the key mediators of fibrosis in the kidney and in other organs (5). Once activated, fibroblasts are designated to be myofibroblasts. Myofibroblasts are the predominant type of interstitial cell in the fibrotic kidney (6) and have been suggested to be the major source of the extracellular matrix (ECM) components that accumulate during renal fibrosis. Myofibroblasts appear *de novo* in areas of fibrosis in response to stimuli such as transforming growth factor (TGF)-β1 (7).

TGF-β1 is a ubiquitous cytokine belonging to the TGF-β superfamily (8). TGF-β1 transduces signaling through a transmembrane receptor serine/threonine complex that comprises the type I and type II receptor kinases. Once TGF-β1 binds to the constitutively active type II receptor, the type I receptor kinase, activin receptor-like kinase, is subsequently recruited and activated by TGF-β type II receptor-mediated phosphorylation. Phosphorylation of serine/threonine residues in the type I receptor kinase subsequently phosphorylates the major downstream signaling mediator proteins, Smad2 and Smad3. Phosphorylated Smad2 (pSmad2) and Smad3 (pSmad3) form a complex with Smad4. This complex translocates into the nucleus and regulates the transcription of fibrosis-associated genes (9). Deregulation of TGF-β 1 has been implicated in the pathogenesis of various diseases, including fibrosis, atherosclerosis and cancer (10). It has been indicated that TGF-β1 acts as a potent fibrogenic cytokine, evoking pathological fibrosis in various organs, including the kidney (11).

Asiatic acid (AA) is one of the triterpenoid compounds present in *Centella asiatica*. A number of studies have shown that AA demonstrates a variety of antitumor (12) and neuroprotective (13) pharmacological effects. In particular, AA has been shown to combat liver fibrosis (14); however, whether AA is able to inhibit renal fibrosis has not yet been elucidated. Therefore, the aim of the present study was to investigate whether AA has an antifibrotic effect in the kidneys of mice with ureteral obstruction, a well-established model of interstitial fibrosis, and to explore any underlying mechanisms.

## Materials and methods

### Animals

Male C57BL6 mice (weight, 18–20 g) were housed at the Experimental Animal Center of Wuhan University (Wuhan, China), at a constant temperature and with a regular light-dark cycle. Food and water were provided *ad libitum*. All surgical and experimental procedures were approved by the Institutional Animal Care and Use Committee of Wuhan University. Efforts were made to minimize any animal suffering and the number of animals used throughout the experiment.

### Agents and antibodies

The purified natural product AA (96%) was obtained from Shanghai Yuanye Biotechnology Co., Ltd. (Shanghai, China) and was used as described in the following sections. The goat polyclonal anti-fibronectin (sc-6952) and anti-p-Smad2/3 (sc-11769) antibodies, and the rabbit polyclonal anti-TGF-β1 (sc-146) antibody was purchased from Santa Cruz Biotechnology, Inc. (Santa Cruz, CA, USA). The rabbit polyclonal anti-collagen III (BA0326) and the mouse monoclonal anti-α-smooth muscle actin (SMA) antibodies (BM0002) were purchased from Boshide Bioengineering Co., Ltd. (Wuhan, China).

### Experimental groups and treatments

Twenty-five male C57BL6 mice were randomly assigned into five groups, with five mice per group, as follows: (i) sham-surgery (Sh); (ii) unilateral ureteral obstruction plus vehicle (UUO+V); (iii) UUO plus 1 mg/kg body weight AA (UUO+A1); (iv) UUO plus 4 mg/kg body weight AA (UUO+A2); and (v) UUO plus 16 mg/kg body weight AA (UUO+A3). The AA dosages used in this study were chosen on the basis of a previous study (15) and the UUO was conducted using an established procedure (16). Briefly, under sodium pentobarbital (40–60 mg/kg body weight)-induced anesthesia, complete ureteral obstruction was performed by ligating the left ureter at the level of lower pole of the left kidney using 4-0 sutures, following a left abdominal incision. Sham-surgery mice had their ureters exposed and manipulated, but not ligated. For mice receiving the AA treatment, escalating doses of AA (1, 4, and 16 mg/kg body weight), suspended in 1.2% methyl cellulose (MC) were administered daily by oral gavage from the day subsequent to the surgery for six days. Mice receiving the vehicle treatment were administered 300 μl phosphate-buffered saline (PBS) in 1.2% MC. Mice were sacrificed on the seventh day subsequent to surgery, and the obstructed kidneys were harvested. A sample of the kidney was fixed in 4% buffered paraformaldehyde and was embedded in paraffin for histological and immunohistochemical studies. The remaining kidneys were snap-frozen in liquid nitrogen and stored at −80°C for protein extraction.

### Histological and immunohistochemical examination

Kidney sections from the paraffin-embedded tissues were prepared at a 5-μm thickness, using an established procedure (17). Sections were stained with hematoxylin and eosin (H&E) and Periodic acid-Schiff (PAS) reagents to assess the grade of tubular injury. A further set of sections was stained using the Masson’s trichrome method for identifying interstitial collagen.

Collagen III and fibronectin expression was examined with individual antibodies, while the presence of interstitial myofibroblasts was measured with anti-α-SMA antibody. Paraffin-embedded sections (5 μm) were mounted onto glass slides. The sections were dewaxed in xylene three times for 15 min each, rehydrated in decreasing concentrations of ethanol for 5 min and washed twice in PBS for 10 min. Endogenous peroxidase activity was quenched for 10 min with 3% hydrogen peroxide. Once the sections had been washed in PBS, a blocking step was included, using 5% bovine serum albumin (BSA) in PBS for a total of 30 min. Primary antibodies against specific antigens, as described previously, were then incubated overnight at 4°C in a humidified chamber. Subsequent to returning to room temperature, the sections were washed in PBS for 10 min and the biotin-coupled secondary antibodies were incubated for 1 h at room temperature. The sections were washed in PBS for 10 min, prior to antigen-specific positive cells being visualized by horseradish peroxidase-coupled streptavidin and diaminobenzidine reagents, provided by Boshide Biotechnology Co., Ltd. (Wuhan, China). Brown staining represented a positive result. Ten random, nonoverlapping, high-power (original magnification, ×400) fields of each slide were selected for evaluation and tubular injury was scored on a scale from 0 to 3, as previously described by Fujiu *et al*: 0, absent; 1, mild; 2, moderate and 3, severe (18).

### Western blot analysis

Western blot analysis was performed as described in a previous study (19). In brief, the kidney tissues were homogenized with a polytron homogenizer (IKA GmbH, Königswinter, Germany) in a lysis buffer containing 20 mM Tris (pH 7.5), 150 mM NaCl, 1% Triton X-100, sodium pyrophosphate, β-glycerophosphate, EDTA, Na_3_VO_4_ and leupeptin (Biyuntian, Wuhan, China) on ice. The lysates were centrifuged at 12,000 × g at 4°C for 20 min and the protein concentration was determined using a bicinchoninic acid (BCA) protein assay kit (cat. no. p0012s; Biyuntian). Following this, the lysates were mixed with 5X sodium dodecyl sulfate (SDS) loading buffer (125 mM Tris-HCl, 4% SDS, 20% glycerol, 100 mM dithiothreitol (DTT) and 0.2% bromophenol blue). Samples were heated at 95°C for ~5 min prior to loading and the supernatants (40 μg protein/lane) were subsequently separated by SDS-polyacrylamide gel electrophoresis (PAGE) on a 12% acrylamide gel. The proteins were then electrotransferred to a nitrocellulose membrane (Millipore, Billerica, MA, USA) in transfer buffer containing 48 mM Tris-HCl, 39 mM glycine, 0.037% SDS and 20% methanol at 4°C for 1 h. Nonspecific binding to the membrane was blocked for 1 h at room temperature with 5% non-fat milk in Tris-buffered saline (TBS) buffer (20 mM Tris-HCl, 150 mM NaCl and 0.1% Tween 20) or 5% BSA. The membranes were then incubated overnight at 4°C with various primary antibodies in a blocking buffer containing 5% milk, at the dilutions specified by the manufacturers. Following this, the membranes were incubated with horseradish peroxidase-conjugated secondary antibodies, developed with the enhanced chemiluminescence plus detection system (Amersham, Buckinghamshire, UK) (20).

### Statistical analysis

Data are presented as the mean ± standard deviation. One-way analysis of variance (ANOVA) and the Student-Newman-Keuls test were used for quantitative data, while Kruskal-Wallis ANOVA was used for abnormally distributed data. Statistical analyses of the data were performed using the Graphpad Prism^®^ software package, version 5.0 (Graphpad Software, Inc., La Jolla, CA, USA). P<0.05 was considered to indicate a statistically significant difference.

## Results

### AA attenuates tubular injury

**Figure 1:**
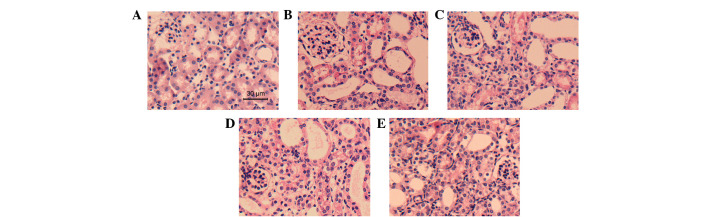
Representative photomicrographs of the histopathological examinations of the (A) sham-surgery; (B) unilateral ureteral obstruction plus vehicle (UUO+V) ; (C) UUO plus 1 mg/kg body weight asiatic acid (AA) ; (D) UUO plus 4 mg/kg body weight AA and (E) UUO plus 16 mg/kg body weight AA groups (hematoxylin and eosin staining; scale bar, 30 μm; magnification, ×400).

**Figure 2:**
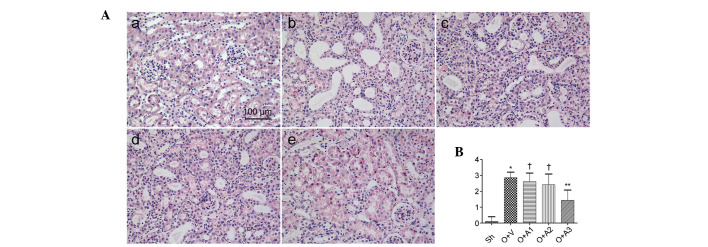
(A) Representative photomicrographs of the histopathological examinations of the (a) sham-surgery (Sh) ; (b) unilateral ureteral obstruction (UUO) plus vehicle (O+V) ; (c) UUO plus 1 mg/kg body weight asiatic acid (AA) (O+A1) ; (d) UUO plus 4 mg/kg body weight AA (O+A2) and (e) UUO plus 16 mg/kg body weight AA (O+A3) groups (Periodic acid-Schiff staining; scale bar, 100 μm). (B) Semiquantitative analysis of tubular injury. All data are presented as the mean ± standard deviation (n=5 per group). ^*^P<0.05 compared with the Sh group; †P>0.05 compared with the O+V group; ^**^P<0.05 compared with the O+V group (magnification, ×400).

Seven days subsequent to the ureteral obstruction, the effect of AA on the suppression of tubular injury was examined. H&E- and PAS-stained micrographs of the kidney are shown in Figs. 1 and 2, respectively. A significantly greater amount of tubular damage was observed in the UUO+V group compared with the Sh group (2.86±0.35 versus 0.10±0.30, respectively; P<0.05); however, this damage was alleviated in the UUO+A3 group (1.44±0.64 versus 2.86±0.35, P<0.05). The tubular damage was not significantly alleviated in the UUO+A1 (2.62±0.53 versus 2.86±0.35, P>0.05) or UUO+A2 (2.42±0.67 versus 2.86±0.35, P>0.05) groups.

### AA attenuates interstitial fibrosis

**Figure 3:**
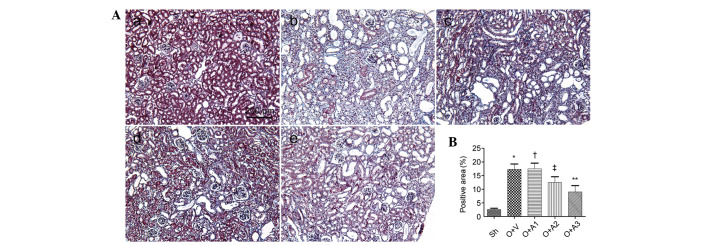
(A) Representative photomicrographs of the histopathological examinations of the (a) sham-surgery (Sh); (b) unilateral ureteral obstruction (UUO) plus vehicle (O+V); (c) UUO plus 1 mg/kg body weight asiatic acid (AA) (O+A1); (d) UUO plus 4 mg/kg body weight AA (O+A2) and (e) UUO plus 16 mg/kg body weight AA (O+A3) groups (Masson’s trichrome staining; scale bar, 200 μm). (B) Semiquantitative histogram for positive area of collagen. Data are presented as the mean ± standard deviation (n=5 per group).^*^P<0.05 compared with the Sh group; †P>0.05 compared with the O+V group; ‡,^**^P<0.05 compared with the O+V group (magnification, ×200).

**Figure 4:**
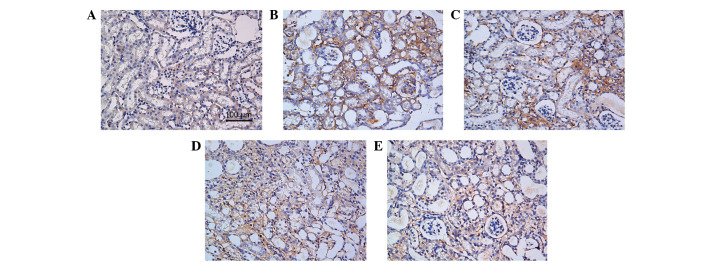
Representative photomicrographs showing collagen III staining in the (A) sham-surgery; (B) unilateral ureteral obstruction plus vehicle (UUO+V); (C) UUO plus 1 mg/kg body weight asiatic acid (AA) ; (D) UUO plus 4 mg/kg body weight AA and (E) UUO plus 16 mg/kg body weight AA groups (Masson’s trichrome staining; scale bar, 100 μm; magnification, ×400).

**Figure 5:**
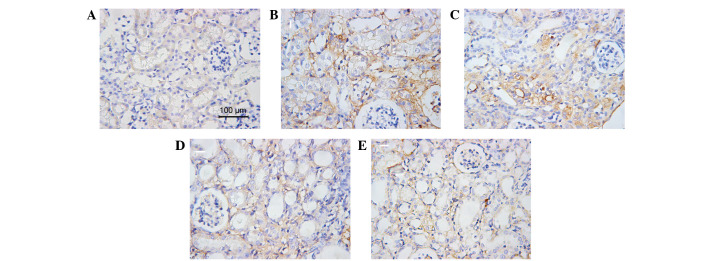
Representative photomicrographs showing fibronectin staining in the (A) sham-surgery; (B) unilateral ureteral obstruction plus vehicle (UUO+V); (C) UUO plus 1 mg/kg body weight asiatic acid (AA); (D) UUO plus 4 mg/kg body weight AA and (E) UUO plus 16 mg/kg body weight AA groups (Masson’s trichrome staining; scale bar, 100 μm; magnification, ×400).

Seven days subsequent to the ureteral obstruction, the effect of AA on the suppression of interstitial fibrosis was examined. Masson’s trichrome-stained micrographs of the kidney are shown in Fig. 3. A significantly greater amount of interstitial fibrosis was observed in the UUO+V group compared with the Sh group (17.28±1.98 versus 2.56±0.46, respectively; P<0.05); however, this fibrosis was alleviated in the UUO+A2 (12.51±2.09 versus 17.28±1.98, P<0.05) and UUO+A3 (9.03±2.31 versus 17.28±1.98, P<0.05) groups. Interstital fibrosis was not alleviated in the UUO+A1 group (17.48±2.08 versus 17.28±1.98, P>0.05). The anti-fibrotic effect of AA was also shown by histopathological staining of collagen III and fibronectin (Figs. 4 and 5).

### AA decreases the accumulation of myofibroblasts

**Figure 6:**
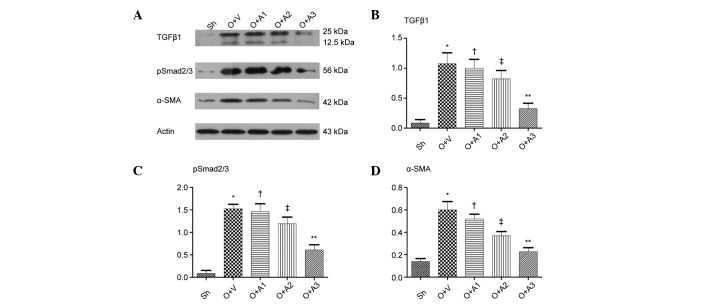
(A) Representative western blot gels for transforming growth factor (TGF)-β1, phosphorylated (p) Smad2/3 and α-smooth muscle actin (SMA). (B–D) Semiquantitative histograms for TGF-β1 (B), pSmad2/3 (C) and α-SMA (D). Sh, sham-surgery group; O+V, unilateral ureteral obstruction (UUO) plus vehicle; O+A1, UUO plus 1 mg/kg body weight asiatic acid (AA); O+A2, UUO plus 4 mg/kg body weight AA; O+A3, UUO plus 16 mg/kg body weight AA. ^*^P<0.05 compared with the Sh group; †P>0.05 compared with the O+V group; ‡,^**^P<0.05 compared with the O+V group.

As shown in Fig. 6, western blot analysis demonstrated that ureteral obstruction resulted in a marked increase in α-SMA expression in the UUO+V group mice as compared with the Sh group mice (0.60±0.07 versus 0.14±0.03, respectively; P<0.05). Compared with the UUO+V group, the UUO+A2 and UUO+A3 groups demonstrated significant reductions in α-SMA expression in renal tissue (0.37±0.04 and 0.23±0.04, respectively, versus 0.60±0.07, P<0.05). There was no significant difference between the UUO+A1 and the UUO+V groups (0.52±0.04 versus 0.60±0.07, respectively; P>0.05).

### AA inhibits the TGFβ1 pathway

As shown in Fig. 6, western blot analysis demonstrated that ureteral obstruction resulted in a marked increase in the level of TGF-β1 in the UUO+V group as compared with Sh group (1.08±0.18 versus 0.09±0.06, respectively; P<0.05). Compared with the UUO+V group, the UUO+A2 and UUO+A3 groups demonstrated significant reductions in TGF-β1 expression in renal tissue (0.82±0.14 and 0.33±0.09, respectively, versus 1.08±0.18; P<0.05); however, there was no significant difference in the TGF-β1 expression between the UUO+A1 and UUO+V groups (1.00±0.15 versus 1.08±0.18, respectively; P>0.05). Ureteral obstruction also led to a significant increase in p-Smad2/3 levels in UUO+V mice as compared with the Sh group mice (1.52±0.10 versus 0.09±0.07, respectively; P<0.05). The UUO+A2 and UUO+A3 groups demonstrated significant reductions in p-Smad2/3 levels in the renal tissue compared with the UUO+V group, (1.19±0.15 and 0.61±0.11 versus 1.52±0.10, respectively; P<0.05). However, there was no significant difference between the UUO+A1 and UUO+V groups (1.47±0.17 versus 1.52±0.10, P>0.05).

## Discussion

In CKD, progressive renal tissue injury leads to an irreversible reduction in renal function. At present, there are no specific treatments for CKD, which ultimately progresses into ESRD. Renal replacement therapy (RRT) is the only therapeutic option available. The main pathological feature of CKD is interstitial fibrosis, and this is positively correlated with the prognosis of the patient; therefore, the attenuation of renal interstitial fibrosis presents a therapeutic option for patients with CKD. A previous study indicated that AA inhibited liver fibrosis induced by CCl_4_(14), while the present study demonstrated that AA was able to attenuate renal fibrosis in mice with UUO.

Tubular epithelial cells are the predominant component of renal parenchyma and are the primary target in a variety of injuries. Depending on the severity and duration of the injury, tubular cells exhibit a wide range of responses (3). In patients with CKD, the histopathological presentation of tubular damage is often characterized by tubular atrophy, most likely as a consequence of apoptosis and epithelial-mesenchymal transition (EMT) (8). While activated and injured tubules compromise renal function, those tubules may also be involved in the process of interstitial fibrosis, by producing profibrotic and proinflammatory cytokines (21). The present study revealed that high-dosage AA alleviated tubular injury, although similar effects were not observed with lower and intermediate dosages. AA may, therefore, preserve tubular function and halt the progression of interstitial fibrosis.

The ECM is mainly composed of collagen and proteoglycans (22). In the glomerular and tubulointerstitial compartments, there is a small amount of ECM, which constitutes the framework structure of the renal interstitium. Abnormal deposition of ECM is one of the main pathological changes observed following UUO, and leads to a decrease in the diffusion of oxygen (23). It also contributes to abnormal intercellular signaling, leading to a vicious cycle of ECM deposition (24,25). In the present study, Masson’s trichrome staining revealed that small quantities of ECM were present in the normal kidneys, predominantly in the glomerular basement membrane and the peritubular blood vessels. By contrast, UUO induced the accumulation of a large volume of ECM in the interstitium. Whereas low-dosage AA did not decrease the levels of ECM, intermediate and high dosages of AA resulted in marked reductions. Similar results were observed by the immunohistochemical examination of collagen III and fibronectin. This indicated that AA decreased the volume of ECM, which rescued normal tubulointerstitial architecture and function.

During fibrosis, interstitial myofibroblasts are the main cells producing ECM (26). Although the source of the myofibroblasts remains debated, a strategy that reduces myofibroblast recruitment and inhibits their proliferation and activation is a therapeutic option. Mature myofibroblasts are tissue-contracting cells. It has been shown that biomechanical forces are crucial for cell differentiation and are major triggers for the induction of a myofibroblast phenotype (27). α-SMA is a biomarker of activated myofibroblasts and α-SMA levels represent and correlate with the progression of CKD. The majority of antifibrotic interventions have been shown to be correlated with a reduction in the number of α-SMA-expressing cells (7). The present study showed that intermediate and high doses of AA decreased the expression of α-SMA, although low-dosage AA failed to produce a significant effect. This indicated that AA was able to alleviate renal fibrosis by inhibiting myofibroblast recruitment and activation.

TGF-β1 has been shown to be a central factor in many pathological events associated with CKD progression (28), and contributes to tubular loss, fibroblast recruitment, proliferation and activation and excessive ECM accumulation (2,29). It has been shown that targeting TGF-β1 signaling produces encouraging results (29). Therefore, the present study examined whether AA was able to influence this pathway. The results showed that intermediate and high doses of AA decreased TGF-β1 levels and reduced the phosphorylation of Smad2/3, a downstream meditator of TGF-β1. This finding indicated that AA decreased interstitial fibrosis by affecting the Smad-dependent TGF-β1 signal transduction.

In conclusion, the results of the present study provided *in vivo* evidence that AA was able to attenuate renal tubulointerstitial fibrosis in a dose-dependent manner. The effects of AA may have been mediated by the inhibition of Smad-dependent TGF-β1 signaling which, in turn, attenuated tubular injury and fibroblast recruitment, proliferation and activation.
